# 

**Published:** 1875-04-15

**Authors:** 


*ORIGINAL COMMUNICATIONS:*

Belladonna *vs.* Opium; Administration of Chloroform during Sleep; Unusual Number of Worms. Notes from Practice. By J. J. Schneck, M.D. .......... 207

The Recurrence of Measles. By James I. Tucker, M.D. .......... 209

An Interesting Surgical Case. By H. W. Sawtelle, M.D. .......... 209

*TRANSLATIONS:*

Recent Progress of Ophthalmology in France. By F. J. Huse, M.D. .......... 211

The Innervation of the Heart. By Prof. G. See. Translated by H. M. Bannister, M.D. .......... 215

*EDITORIAL DEPARTMENT:*

Association of American Medical Editors .......... 225

Public Medical Instruction .......... 225

*CORRESPONDENCE:*

A Letter from Paris. By C. C. Matteson, M.D. 226

*GLEANINGS FROM OUR EXCHANGES:*

Biographical Sketch of the late Dr. Rush .......... 229

On the Merits, Real and Comparative, of Amputations in the Lower Third of the Leg, with Suggestions as to the Prospective Value of Periosteal Flaps .......... 231

Nyphomania and Vaginismus Caused by Anal Fissure .......... 233

